# Evolution of Food Fermentation Processes and the Use of Multi-Omics in Deciphering the Roles of the Microbiota

**DOI:** 10.3390/foods10112861

**Published:** 2021-11-18

**Authors:** Mohamed Mannaa, Gil Han, Young-Su Seo, Inmyoung Park

**Affiliations:** 1 Department of Integrated Biological Science, Pusan National University, Busan 46241, Korea; mannaa@cu.edu.eg (M.M.); croone@pusan.ac.kr (G.H.); 2 Department of Plant Pathology, Cairo University, Giza 12613, Egypt; 3 School of Culinary Arts, Youngsan University, Busan 48015, Korea

**Keywords:** fermented food, microbiome, multi-omics

## Abstract

Food fermentation has been practised since ancient times to improve sensory properties and food preservation. This review discusses the process of fermentation, which has undergone remarkable improvement over the years, from relying on natural microbes and spontaneous fermentation to back-slopping and the use of starter cultures. Modern biotechnological approaches, including genome editing using CRISPR/Cas9, have been investigated and hold promise for improving the fermentation process. The invention of next-generation sequencing techniques and the rise of meta-omics tools have advanced our knowledge on the characterisation of microbiomes involved in food fermentation and their functional roles. The contribution and potential advantages of meta-omics technologies in understanding the process of fermentation and examples of recent studies utilising multi-omics approaches for studying food-fermentation microbiomes are reviewed. Recent technological advances in studying food fermentation have provided insights into the ancient wisdom in the practice of food fermentation, such as the choice of substrates and fermentation conditions leading to desirable properties. This review aims to stimulate research on the process of fermentation and the associated microbiomes to produce fermented food efficiently and sustainably. Prospects and the usefulness of recent advances in molecular tools and integrated multi-omics approaches are highlighted.

## 1. Introduction

The art of fermentation is as old as the human civilisation on earth, as it was traditionally developed by ancient societies for food preservation during harsh seasons, for ritual feasts, and to enhance the sensory quality of food [1]. Historical records reveal that fermentation of several substrates, including milk and cereal, is indigenous to many parts of the world. The earliest form of fermentation was discovered by analysing the stone mortars from the Natufian burial sites of a semi-sedentary foraging population, providing archaeological evidence for beer brewing from cereals dating back 13,000 years [2]. In ancient Egypt, dairy products, fermented bread, and beer were dietary staples [3]. In China, chemical analysis of ancient pottery jars indicate the existence of fermented products of rice, honey, and fruits as early as the seventh millennium B.C. [4].

Fermentation was an integral part of other ancient civilisations; examples include beer brewing in Babylonia, soy sauce production in East Asia, and fruit fermentation in Greece (Greeks attributed ‘Dionysos’ as the god of fruit fermentation) [5]. The most ubiquitous type of fermented food is yoghurt (made from milk), which was produced and consumed throughout the Middle East and Europe and has become a major component of the human diet worldwide [6]. In East Asia, a series of fermented food products, mainly based on rice, soybean, vegetables, and fish, have been developed and are still produced and consumed on a daily basis; examples of such products include Korean kimchi and Japanese natto, which have gained popularity worldwide owing to their unique taste and proven health benefits [7].

Fermentation continues to be practised due to the evidence of extended shelf life and improved organoleptic properties of fermented foods. There is a wide variation in fermented foods and drinks prepared and consumed worldwide, although we lack detailed knowledge of the microbial properties underlying such variation [5]. In the past, fermentation was based on naturally occurring microbes in the food substrate that were affected largely by the surrounding condition and environment, leading to the characteristics of the fermented products according to their geographical location. With the growing global attention and the increasing demand for fermented products, merged with the increased awareness of food safety aspects, standardisation of the process was necessary, which led to industrial control of the production procedures, such as the use of starter cultures and the control of fermentation protocols on an industrial scale [8].

The first model for understanding microbial roles in food fermentation was created when the role of the fungus *Aspergillus oryzae* in the preparation of koji was discovered by the German scientist Korschelt in 1878 [9]. This discovery was followed by the identification of various additional fermentation microbes and starter cultures, reaching the recent advancement in molecular biology techniques, next-generation sequencing (NGS), multi-omics and bioinformatics tools, and advanced statistical approaches. These advancements have revealed the microbiome composition of fermented foods and the complete genome sequence of biotechnologically important microbes. These findings have enabled a thorough understanding of the fermentation process and microbial diversity, roles, and metabolic pathways and outcomes, leading to great development in the fermentation industry [9].

Human gut microbiome research has revealed the link between the gut microbiome and different aspects of human health and diseases. This finding has necessitated studies on fermented foods and their roles in enhancing the microbiome. This review aims to discuss the evolution of the fermentation process and the contribution of modern biotechnological tools in improving the process of food fermentation. Furthermore, the modern advancements in multi-omics approaches are reviewed along with their application in studying the fermentation-associated microbiome, microbial interactions within the fermented food ecosystem, and microbial roles in imparting fermented foods and beverages with unique properties. Finally, the future perspectives on food fermentation considering modern innovative approaches are highlighted.

## 2. Classification of Major Types of Fermented Foods and Beverages

**Figure 1:**
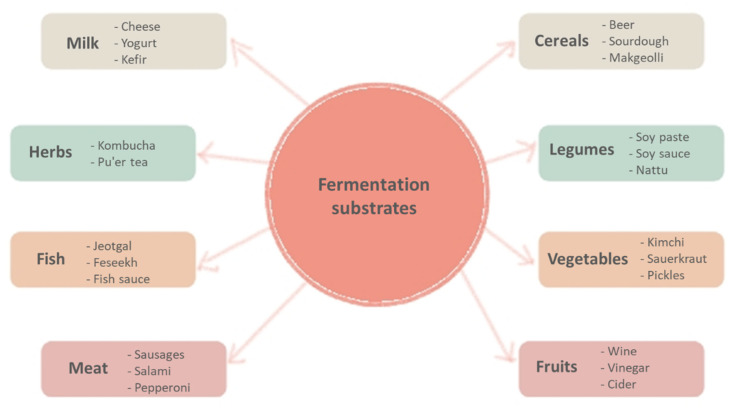
Common fermentation substrates and produced fermented foods and beverages.

Fermentation involves the action of enzymes and catalysts derived from microorganisms such as bacteria, yeast, and moulds for the chemical transformation of the complex organic compounds in the substrate into simpler, bioactive, functional, and nutritious compounds [10]. There are several classification methods for fermented foods and beverages, mainly based on the substrate category used, such as fermented milk, cereal, legumes, vegetables, fruits, meat, fish, and herbs [11]. The various combinations of different types of food substrates and the involved fermentation microbiota give rise to thousands of fermented products worldwide; the main examples of common fermented foods and beverages and the main fermenting microbes are summarised in Figure 1.

Fermentation can also be categorised, according to the main biochemical pathway, into four basic categories: alcoholic, lactic, acetic, and alkali fermentation (Table 1) [12]. In alcoholic fermentation, the sugars in the substrate are converted into alcohol and carbon dioxide; examples of such fermentation include the production of bread, beer, and wine. Yeast is the predominant microbe responsible for this type of fermentation. In lactic fermentation, sugars are converted into lactic acid, as in the case of yoghurt, kimchi, and fermented cereals. Lactic acid bacteria (LAB) are mainly responsible for this type of fermentation [13]. In acetic fermentation, organic compounds such as alcohols and sugars in the substrate are converted into acetic acid by bacteria mainly belonging to the genus *Acetobacter*, as in the case of production of water kefir, kombucha, cocoa, acidic beer, and vinegar [14]. Organic acids that are microbially produced during the fermentation of several fermented foods and beverages play key roles in determining the quality and safety aspects of the products. For example, propionic acid, produced by *Propionibacterium*, and glucuronic acid, produced mainly by *Gluconacetobacter*, impart kombucha with antioxidant properties and strong antimicrobial activity against harmful microbes [15]. The lactic acid bacteria could also be classified into two main physiological groups depending on the fermentation pathway, the homofermentative and heterofermentative. The distinction is in the main product of the fermentation of sugars being primarily lactic acid in the homofermentative and lactic acid, CO_2_, acetic acid and/or ethanol in the heterofermentative group [16].

In alkali fermentation, the proteins in the substate are hydrolysed into amino acids and peptides, releasing ammonia, which elevates the pH (8–9), inhibiting spoilage-associated microbes. Ammonia produced during alkaline fermentation (involved in the preparation of Japanese nattu and African fermented legumes and eggs) is responsible for a strong umami flavour and aroma. Microbes responsible for alkaline fermentation mainly belong to *Bacillus* spp. and coagulase-negative *Staphylococcus*, which can produce extracellular proteinase for protein hydrolysis [17,18]. Unlike fermented products that depend on a specific group of microbes for fermentation, there are fermented foods and beverages, such as Korean doenjang and kombucha, that pass through different stages of fermentation, in which different types of microbes are responsible for the multi-step fermentation process [19].

Steinkraus [20] proposed a seven-category classification of fermented foods and beverages that predicts the involved microorganisms and the changes (chemical, physical, and nutritive) occurring during fermentation. In this classification, textured vegetable-protein meat substitutes such as Indonesian tempe, high salt/meat-flavoured amino acid/peptide sauces, fermented paste such as fish sauce and miso, and leavened and sourdough breads were added as separate categories to the previously described general classification [20].

## 3. Evolution of the Process of Fermentation over the Years

### 3.1. Spontaneous Fermentation and Back-Slopping

Fermented foods and beverages have traditionally been produced by relying on the microbiota naturally occurring on the food substrate. Spontaneous fermentation dependent on autochthonous microbes was the main method for producing fermented food and beverages throughout history, and it remains a mainstay method in domestic, small-scale, and household settings [21]. In this type of fermentation, the conditions are adjusted to allow for the growth of desirable fermentation microbes that impart unique sensory properties to the product and prevent the growth of spoilage-associated microbes [22]. Fermentation conditions often need to be adjusted—for example, creating anaerobic conditions is necessary for the production of pickles; the composition of ingredients may also need to be adjusted (e.g., by adding salt or vinegar during fermentation) to suppress competing undesirable microflora [23]. Many types of fermented foods, such as sauerkraut and kimchi, are still produced using spontaneous approaches without the use of starter cultures, especially in small-scale settings and in developing countries, as the process depends completely on enhancing the growth of microbes available on the substrate raw materials [12].

The start of the fermentation process may involve transferring a small amount of a previously successful fermented batch into fresh ingredients as an inoculum to facilitate the initial phase of fermentation of the next batch, even without the knowledge of the types of active microbes; this process is called back-slopping [24]. In spontaneous fermentation, a successful process is achieved when the desirable microbes can outcompete and dominate harmful and spoilage-associated microbes because of their adaptability to the substrate and the prevailing fermentation condition. Back-slopping reduces the risk of failure and facilitates the competitive ability of fermentation microbes; repeating the process provides further selection of useful microbes that are best adapted to the food substrate and the fermentation condition, providing the currently available starter cultures [22].

### 3.2. Starter Cultures

With progress in the microbiological techniques of isolation, identification, and microbial preservation, specific starter cultures have been isolated and characterised from fermented foods. These cultures are currently used, especially on the industrial scale, to ensure that the process is controlled and the fermentation outcome is stable for quality and properties. The use of well-defined starter cultures was first adopted to produce beer, alcohol, vinegar, and bread, followed by dairy and meat products [25]. The main role of starter cultures is to accelerate the fermentation process and to convert carbohydrates in the substrate into alcohols and organic acids which act as natural preservatives that restrict the growth of harmful microbes and impart distinct and desirable organoleptic properties to the product. Minimising the risk of foodborne diseases has been confirmed previously in natural conditions and in artificial inoculation with pathogens [26,27]. The release of carbon dioxide by the action of starter cultures is important for the process of fermentation, as it contributes to rising the dough during breadmaking, making the foam of beer and buttermilk, and the formation of eyes in cheese [25].

The starter cultures that are mostly used to produce fermented foods and beverages, particularly acidic fermented products, belong to LAB [25]. Such bacteria include members of *Lactobacillus, Leuconostoc*, *Enterococcus, Streptococcus, Oenococcus*, and *Pediococcus*, and some of them may exert direct beneficial effects on health as live probiotic microbes [28]. Additionally, non-LAB bacteria, such as those belonging to *Bacillus*, *Micrococcaceae*, *Bifidobacterium*, *Propionibacterium*, and *Brachybacterium*, act as a secondary group of microorganisms in the fermentation process [29]. Along with bacteria, yeast and moulds, including several species of *Debaryomyces, Kluyveromyces, Saccharomyces, Aspergillus, Mucor, Penicillium,* and *Rhizopus* species, represent an important part of starter cultures in a variety of fermented foods and beverages, such as cheese and coffee, in which the microbiota significantly affects the appearance and organoleptic properties [30,31]. For wine making, the *Saccharomyces cerevisiae* is traditionally used in the fermentation process. In addition, there is an increasing awareness about the enological characteristics of other non-*Saccharomyces* yeast in imparting the wine with particular flavour and aroma [32].

### 3.3. Starter Cultures of Multiple Strains and Adaptation for Co-Existence

Starter cultures do not always contain a single strain; in many cases, a consortium of different organisms and strains is involved. The model example for the fermented beverages to be covered in this review is kombucha, with a starter culture consortium of multiple species that are well-adapted to co-existence. In the case of kombucha, a fermented, sweetened, black tea-derived beverage, which originated in China thousands of years ago and is currently gaining popularity worldwide for its health-promoting and therapeutic effects, a symbiotic culture of bacteria and yeast (SCOBY) is used to initiate fermentation [33]. Kombucha fermentation comprises three main types of fermentation (i.e., alcoholic, lactic, and acetic) due to the presence of different types of bacteria and yeast co-existing in the medium and responsible for different stages of fermentation; the process is initiated by osmotolerant microbes, and acid-tolerant bacteria prevail and dominate [34].

In this case, the microbes are well-adapted to the substrate and co-exist with other microbes constituting the SCOBY. They act in harmony; the substrate contains sucrose that is first broken down by the action of yeast (*Saccharomyces cerevisiae*) into fructose and glucose, which are then used for the growth of bacteria in the consortium (e.g., *Acetobacter* and *Gluconobacter* spp.) producing various organic acids, such as acetic, gluconic, and glucuronic acids [35]. Yeast in kombucha ferments the sugar into ethanol and CO_2_; ethanol is subsequently oxidised into acetic acid by acetic acid bacteria. These organic acids, along with the alcohols produced by the yeast, act as antimicrobial agents that inhibit the growth of undesirable microbes in kombucha [35]. The levels of polyphenols and flavonoids originally found in black tea increase progressively with fermentation, most likely due to the role of yeast in enzymatically degrading the polyphenols into smaller molecules, increasing the antioxidant activity of kombucha and stimulating the production of bacterial cellulose [36,37].

**Figure 2:**
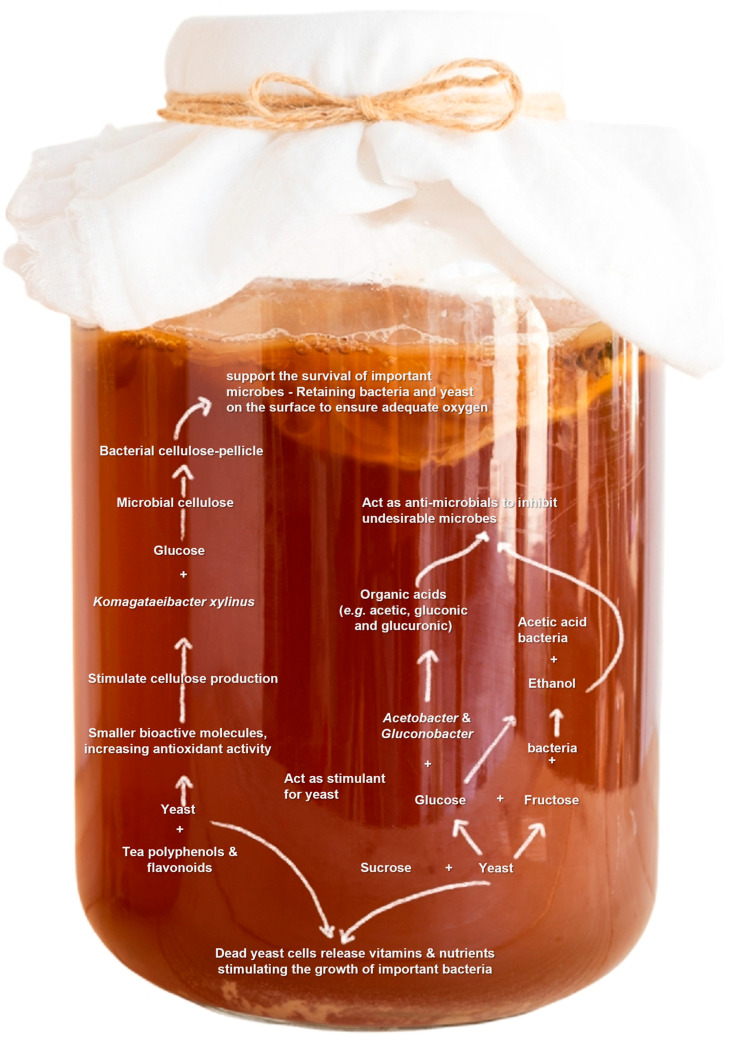
An illustration of the metabolic interplay and functional compatibility of kombucha fermentation microbiota, representing a model for the adaptation and symbiosis of the microbiota in the fermentation ecosystem.

The microbial cellulose produced by *Komagataeibacter xylinus* (formerly *Gluconacetobacter xylinus*) is the base for forming the floating biofilm as the solid phase of kombucha. Formation of this biofilm enhances the association between bacteria and yeast and plays a role in adjusting the fermentation condition to support the survival of important microbial groups by retaining bacteria and yeast on the surface of the liquid to ensure adequate oxygen supply and nutrient diffusion (by forming reticulation) to inhabiting bacteria [38]. The roles of the microbial agents within the kombucha ecosystem are not limited to their biological activity; even after the death of the involved yeast cells, they release vitamins and nutrients, stimulating the growth of important bacteria [34]. This phenomenal co-existence of different interacting microbes constituting the consortium of kombucha fermentation represents a model for fermentation microbiota co-evolution, powerful symbiosis, and ecological system stability; this consortium can tolerate simulated Mars-like environmental conditions and restore their biological activity after exposure [34,39]. Figure 2 illustrates the metabolic interplay and functional compatibility of the kombucha fermentation microbes, indicating their adaptation and strong symbiosis.

### 3.4. Genetic Improvement of Starter Cultures

The fermentation process has further evolved as the use of starter cultures has undergone significant improvement with the advancement of molecular biology techniques. Previously, the selection of starter cultures was based on the screening of many isolates, and those that performed well in fermentation on an industrial scale, yielding end products with acceptable organoleptic characteristics, were selected [40]. Recently, advanced tools allowing for high-throughput screening for specific targeting of genes and metabolic pathways have resulted in the selection of better performing and well-adapted starter cultures for improved fermentation and facilitated the selection of mutants and genetic engineering for superior starter cultures with desired properties [25].

The successful plasmid transformation of *Lactococcus lactis* (formerly *Streptococcus lactis*), an important microorganism for dairy fermentation, using recombinant DNA techniques in 1982, was considered a turning point for using genetic engineering to improve starter cultures for preparing fermented food [41]. Following this advancement, several industrially important LAB, such as *Streptococcus thermophilus* and members of the *Leuconostoc* genus, have been genetic modified to improve traits linked to metabolism, efficiency of proteolysis, and defence against bacteriophages [42]. Infection of starter cultures with bacteriophages is a major concern for dairy fermentation, as this causes significant economic losses due to the rapid accumulation of bacteriophages, leading to the complete termination of acidification and consequent spoilage [43]. Progress in molecular biology has led to the characterisation of bacteriophages coupled with sequencing of the whole genome of *L. lactis*, facilitating understanding of the process of bacteriophage infection and bacterial defence mechanisms. This discovery was translated into constructing strains with bacteriophage components that inhibited phage proliferation and offered significant protection to *L. lactis* [44]. Moreover, genetic and metabolic engineering of LAB opens the way for further utilisation of milk lactose during fermentation with the possibility of generating new useful products (both simple and complex) along with lactic acid, with various beneficial applications [45].

Starter cultures have been generated using recombinant DNA technology for decades and may provide improved fermentation processes and offer better-quality products with desired properties. Despite such potential, none of the developed strains are being used in the industry due to strict governmental regulations and the lack of consumer acceptance of genetically modified food ingredients [46]. Therefore, strain improvement methods without the use of recombinant DNA technology, such as random mutagenesis, directed or adaptive evolution, and dominant selection, together with natural mechanisms such as bacteriophage transduction, natural competence, and conjugation, are widely used in the food industry [47]. Random mutagenesis induced by classical methods (e.g., UV treatment) and subsequent selection of useful variants have been successfully used to generate starter cultures with desired properties [48,49]. However, this method has the disadvantage of causing unintended mutations that might impair the applicability of strains and affect their performance, as evidenced by bacterial whole genome studies [47]. Improved strains qualify as ‘generally regarded as safe’ by the U.S. FDA if they have genetic stability, no foreign DNA, no antibiotic-resistance-marker genes, and no global changes from the parental strain; such improved strains have been generated and registered for use [50]. One example of a registered improved strain as starter culture is the metabolically engineered urea-degrading *Saccharomyces cerevisiae* yeast strain generated to reduce the content of ethyl carbamate, a potent carcinogen in wine; the strain reduces ethyl carbamate in wine by 89.1% and was patented and registered for producing alcoholic beverages [51].

### 3.5. CRISPR/Cas9 Technology for Genetic Improvement of Starter Cultures

The revolutionary novel technology, CRISPR/Cas9 (Clustered Regularly Interspaced Short Palindromic Repeats/CRISPR-associated protein 9), is an extremely precise method of gene editing; it has taken genetic engineering to another level with a wide range of biotechnological applications in many fields, and its discoverers were selected for the Nobel Prize in Chemistry in 2020 [52,53]. Briefly, CRISPR/Cas9 is based on the mechanism of ‘adaptive immunity’, which is naturally found in bacteria and archaea, and comprises two components: the chimeric guide RNA (gRNA) and the RNA-guided DNA endonuclease (e.g., Cas9); the CRISPR/Cas9 toolbox can be used for precise genome editing of any organism [54]. The applications of the toolbox in the food industry are numerous, and it has been applied to improve the strains of starter cultures by producing marker-less, genetically stable strains with improved properties [50].

The technology was first applied in 2013 for genome engineering of *Saccharomyces cerevisiae,* an industrially important yeast and starter strain for several fermented products [55]. Since then, several applications of the CRISPR/Cas9 toolbox to improve the applicability of *S. cerevisiae* have followed. Recently, engineering yeast for the reduction of production of urea, the precursor of ethyl carbamate, and the modulation of glycerol production in wine have been successfully implemented [56,57,58]. The application of the CRISPR/Cas9 toolbox has been explored for improving fermented food and beverage starter microbes along with yeast. Kimchi-associated *Leuconostoc citreum* was engineered using the CRISPR/Cas9 toolbox for elimination of cryptic plasmids and this process was suggested as a food-grade method to develop a safe lactic acid bacterial strain without residual antibiotic markers [59]. Katayama et al. [60] utilised the CRISPR/Cas9 toolbox to develop a functional and versatile genome editing method for efficiently targeting mutagenesis in *A. oryzae*, which is an industrially important filamentous fungus used in Japanese and Korean traditional fermentation [60].

The novel CRISPR technology for gene editing is promising for food-grade applications; it is highly precise, stable, and should be considered outside the scope of genetically modified organisms (GMO) as the modification occurs in nature [50]. However, it still falls into the definition of GMO according to the European Union court, as it concerns organisms made through in vitro mutagenesis [61]. The scientific community is putting effort into the reconsideration of such regulations and the acceptance of the technology in the food industry [62]. Nevertheless, research on improving the properties of starter cultures of fermented food and beverages is critical to improve the quality of products and reduce the potential hazards posed by undesired microbes or their metabolites.

### 3.6. CRISPR-Mediated Microbiome Engineering and Fermentation

Microbial activity in fermented foods and beverages is the main factor responsible for product quality and safety. Hence, the manipulation of the microbial composition, particularly at the initiation of fermentation, is key for controlling the process and shaping the properties of the product. Recent advances in biotechnological tools and the rise of CRISPR-based technologies may not only be involved in genetic improvement of specific strains in starter cultures, as explained earlier, but the technology also has great potential in microbiome engineering [63]. This superior method can target specific groups of undesirable microbes within the fermented food ecosystem and control the microbiota assembly to enhance desirable fermentation microbes, leading to the optimisation of fermented food products [64].

CRISPR-mediated microbiome manipulation has been investigated in several recent studies. The main priority for fermented food and beverage microbiome manipulation is to selectively control the undesirable spoilage-associated microbes or microbes competing with fermentation-desirable microbes to enhance product quality and extend shelf life [64]. Specific targeting of specific individual microbial strains within microbial consortia was previously carried out using the CRISPR/Cas9 toolbox in *Escherichia coli* as a model microbe by targeting specific sequences [65].

**Figure 3:**
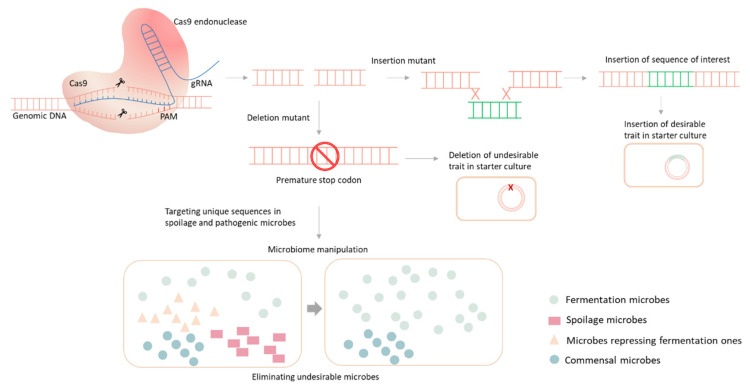
CRISPR-Cas9-mediated gene editing and its possible applications in food fermentation. The precise gene editing can be utilised for improving of the starter culture by deletion of undesirable traits or insertion of desirable traits. CRISPR-Cas9 technology could be utilised for microbiome engineering by targeting unique sequences and selectively eliminate spoilage and undesirable microbes from the community.

Such specific targeting of individual strains in mixed cultures and the differentiation between pathogenic and beneficial microbes was nearly impossible using tailored growth conditions or traditional antibiotics; such targeting allows for further applications by selectively clearing contaminating microorganisms and quantitively controlling the environmental or industrial microbial community composition [65]. The specific DNA sequences responsible for undesirable features that are unique to pathogenic or spoilage microbes, such as virulence factors, antibiotic resistance, or toxin production, can be targeted for elimination [64,66,67]. Although most studies utilising innovative CRISPR-based selective antimicrobial approaches focus on pathogenic microbes, involving selective manipulation of the food-fermentation microbiome by targeting the genotype of spoilage microbes. The different applications and contributions of CRISPR-based technologies in improving food fermentation by targeted gene editing for improvement of starter culture microbiome engineering are shown in Figure 3.

**Figure 4:**
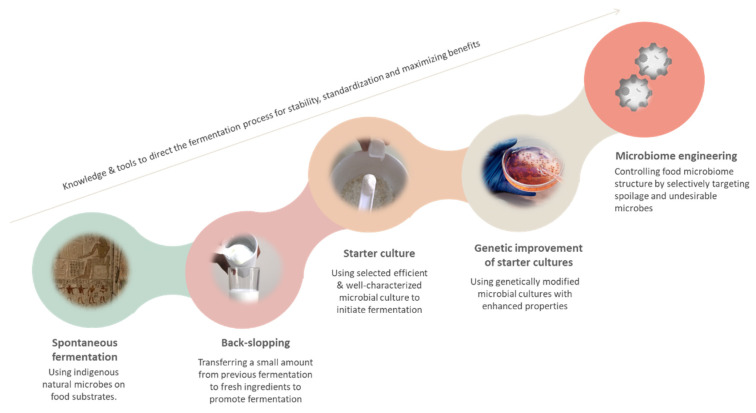
Evolution of the process of fermentation throughout the history, starting from relying on the natural indigenous microbes reaching to the innovative approach of microbiome engineering using advanced technological tools.

CRISPR-based microbiome engineering represents the most recent advancement in the evolution of the food fermentation process, starting with traditional spontaneous fermentation through the use of starter cultures and genetic engineering. The evolution of the food fermentation process is summarised in Figure 4.

## 4. Multi-Omics and Microbiota Dynamics of Food Fermentation

**Figure 5:**
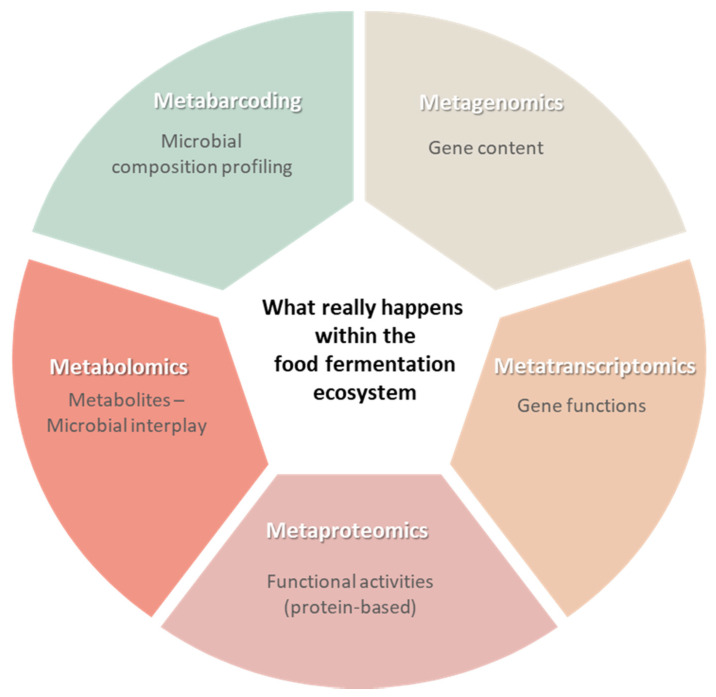
The different tools of the multi-omics analysis and their roles in understanding the food fermentation process, microbiome structure and functional activity profiling.

The continuously interacting microbiota of food fermentation ecosystems, encompassing different types of bacteria, yeast, and fungi, plays a major role in shaping the quality and safety of fermented foods and beverages [68]. Previous studies investigating the microbial composition of fermented products were based on the culture-based traditional plate cultivation method, which failed to provide accurate information about the microbial profiles mainly because of the vast majority of uncultivatable microbes and the presence of viable but not culturable microbes, especially in fermented food ecosystems [21,69]. The recent advancement of the NGS technology, incorporating the collective studies of microbial genomes and metagenomics, meta-transcriptomics, meta-proteomics, and metabolomics to study microbial communities, has enabled accurate identification of the microbial composition of different ecosystems, including fermented food, and the detailed study of the microbe-microbe and microbe-environment interactions within food fermentation ecosystems, involving microbial gene expression, activities, and metabolomic interplay [70]. These nucleic acid and protein-based next-generation approaches have replaced traditional culture-based methods for microbial community profiling and are the cornerstone for understanding the fermentation process in detail and providing opportunities to interfere and manipulate the community composition for improved fermentation processes for safer and better-quality products with desired properties and extended shelf life [71]. Integrated multi-omics analyses and their roles in studying the fermentation microbial ecosystem are summarised in Figure 5.

One of the most extensively applied techniques in recent studies to profile the microbiota within food fermentation is the use of high-throughput sequencing (HTS)-based metabarcoding, by analysing the collective genomic markers by employing universal primers, such as the 16S rRNA and internal transcribed spacer (ITS) regions for bacteria and fungi, respectively [71]. The limitation of this method is that it might fail to identify the microbes involved at the species level, although in many cases the species could be inferred due to the limited number of identified species within many food fermentation genera [71]. Furthermore, this method can only provide qualitative and pseudo-quantitative assessment of the present microbiota that would be expressed as ‘relative abundance’ and this limitation could be overcome by integrating targeted molecular cell enumeration techniques to provide an absolute abundance assessment [72]. Relying on the amplification from DNA templates may limit evaluating the actual active microbial groups, which could be avoided by using RNA-based approaches, including the reverse-transcription of mixed RNA followed by amplification of cDNA that can profile active microbial populations and quantitative PCR for microbial enumeration [73].

A more comprehensive approach to study the fermented food microbiome is the application of metagenomic shotgun DNA-seq, which provides more accurate taxonomic information on the microbial communities of high-complexity samples, allowing for profiling the functional potential by detection of the global gene content and identification of unknown species [74]. The applications of the shotgun DNA-seq approach in studying food matrices include the detection of foodborne pathogens and monitoring changes in the gene content during the fermentation process [71,75,76,77]. Nevertheless, the HTS-based metabarcoding method provides a powerful tool to profile the microbiota of food fermentation and has been successfully applied to investigate different types of food fermentation products and to monitor the microbial dynamics and possible alterations in the microbiota by adjusting the external perturbations such as fermentation conditions, ingredients, and sampling points [78]. A comprehensive review of studies utilising amplicon based HTS was reviewed by Ferrocino and Cocolin, 2017 [71]. Among the applications of the HTS-based metabarcoding approach, the microbiota involved in the process of fermentation and the influence of manipulating the fermentation ingredients on the microbial community structure have been investigated.

### 4.1. Examples on the Use of Meta-Omics to Study Microbial Dynamics of Food Fermentation in Recent Studies

Among the model examples for studies involving HTS-based metabarcoding approach to profile the microbial community structure and evaluate the influence of fermentation ingredients on the microbial community composition and functional potential that will be covered in this review are kimchi, fermented soy products, and kombucha. Kimchi is the most famous Korean fermented food that has gained worldwide popularity owing to its health-promoting properties [79]. Several studies have investigated the origins of the microbial community and their dynamics in kimchi, as well as the identity of main microbes involved in the fermentation process [80,81]. The main microbes of kimchi fermentation are LAB that initiate fermentation by metabolising vegetable sugars into lactic acid, which reduces the pH and limits the growth of most microbes [82]. The dominant microbes in kimchi are members of the *Leuconostoc*, *Lactobacillus*, and *Weissella* genera. During the initial fermentation stage, *Leuconostoc mesenteroides* usually dominates the microbial community, and *Lactobacillus sakei* and *Weissella koreensis* begin to dominate at the optimum-ripening and over-ripening fermentation stages; although the latter are important for kimchi fermentation, their rapid growth and activity promote acidic deterioration, decrease the fresh flavour, and reduce the shelf life [82,83,84,85].

Mannaa et al. [85] utilised HTS-based metabarcoding to investigate the influence of incorporating gizzard shad fish (*Konosirus punctatus*) during kimchi fermentation on the microbial and chemical composition. The purpose of this study was to evaluate a practice that is traditionally adopted in the coastal cities of Korea, where fish is added during kimchi fermentation, and this type of kimchi has a refreshing taste and a relatively extended shelf life [86,87]. This study revealed that adding gizzard shad fish during kimchi fermentation had a positive effect on the composition of chemicals and the microbiota by reducing the growth of *Lactobacillus sakei,* which is linked to the rapid acidic deterioration of kimchi, and by promoting the growth of *Leuconostoc rapi,* which is known for its health-promoting and taste-improving properties owing to the production of the antioxidant mannitol, contributing to the refreshing flavour and desirable characteristics of kimchi [85,88,89]. These results shed light on the inherited wisdom in preparing traditional fermented foods by combining specific substrates that provide and enhance the growth of desirable microbes and suppress spoilage. The formulated ingredients in kimchi act as a source of specific microbes and may exert a selective action on the desired microbes by their potential antibiotic effect (e.g., garlic and red pepper powder) against certain microbial species and by adjusting the physicochemical conditions of the fermentation ecosystem [90,91,92]. Understanding the fermentation process and adjusting the optimum conditions require investigating the roles of fermentation ingredients in shaping the fermentation microbiome.

In other common Korean fermented products, Korean fermented soy paste (doenjang) and soy sauce (gangjang), manipulating the ingredients during fermentation can cause significant changes in the microbial composition and lead to changes in the properties of the product. Fermentation of doenjang and gangjang is based on two steps, starting with meju, whereby dried soybean blocks are fermented spontaneously using naturally occurring populations of fungi and bacteria. The fermented dry mouldy blocks are then subjected to a second long-term fermentation to produce the solid paste, doenjang, and liquid gangjang [93]. The process of fermentation is mostly spontaneous and carried out under non-sterile conditions based on natural microbes from the substrates used. Therefore, there is wide variation in the microbial composition of the product, and the substrates play a major role in controlling the taste, safety, and quality aspects [94].

Mannaa et al. [95] investigated the influence of incorporating fresh coriander during fermentation to produce gangjang using an HTS-based 16S rRNA metabarcoding and reported significant shifts in the microbial composition compared to the control group. Adding coriander resulted in a significant reduction (~45% reduction) in the relative abundance of *Chromohalobacter beijerinckii*, which dominated the microbial community in the control group. Reduction in *C. beijerinckii* is considered beneficial for gangjang as it is responsible for the increase in the levels of biogenic amines, such as histamine, putrescine, and tyramine, which are considered potential health risk factors in fermented salty products and should be minimised. This study combined the metabolomic analysis using ^1^H-NMR to evaluate the content of biogenic amines produced in the gangjang, which revealed a significant reduction in the levels of these biogenic amines. This study demonstrates the advantage of integrating multi-omics tools to evaluate the effect of ingredients on the fermentation process and the end product, by suggesting that adding coriander during fermentation has a positive influence on the quality and health of the product.

Similarly, the effect of adding different types of herbs during fermentation for the production of doenjang was investigated using HTS-based 16S rRNA metabarcoding and metabolomic studies [96,97]. The results indicated that the incorporation of herbs, especially peppermint and Korean mint, during doenjang fermentation had a positive effect on the microbial community structure as the levels of undesirable microbes, such as *Sphingobacterium* and *Pantoea*, were significantly reduced, while potentially beneficial bioactive metabolite-producing microbes, such as *Saccharopolyspora* and *Buttiauxella*, were present at significantly higher levels [97]. These results were further confirmed and were consistent with those of a recent study that utilised primary and secondary metabolome analyses to evaluate the effect of adding herbs on doenjang properties. The results indicated that adding herbs caused significant shifts in the metabolic composition of both the primary and secondary metabolites, with a more profound positive effect on the secondary metabolites, particularly with peppermint and Korean mint treatments; the levels of isoflavones, soyasaponins, and lysophospholipids were significantly increased along with significantly higher antioxidant capacity compared to doenjang made without herbs [96].

Metagenomic approaches have been applied to study the microbial composition of kombucha by combining whole metagenome sequencing, 16S rRNA and internal transcribed spacer-1 amplicon analysis. The results indicated that *Komagataeibacter* and *Zygosaccharomyces* were dominant at different fermentation times. Moreover, functional complementarity was observed between both microbial groups which explains the sustainability of the kombucha ecosystem by ensuring microbial metabolic cross talks [98]. Such mutualistic metabolic interplay (briefly described above) between the microbial groups comprising of the kombucha consortia may explain their stability and ability to tolerate harsh environments. The ecological resilience of the kombucha microbiota to long-term exposure to the extremely harsh conditions of the Mars-like conditions in a low Earth orbit was confirmed by shotgun metagenomic analysis as the core microbial structure was maintained, and there were no significant changes in the community functions, such as the ability to produce cellulose-based pellicles, allowing for the survival of the microbial community under extra-terrestrial conditions [99].

### 4.2. Functional Activity of the Food Fermentation Microbiome

Several studies have combined meta-transcriptomic analysis, which targets actively expressed genes under specific conditions, with meta-barcoding to decipher the core functions of the detected microbiota that are associated with metabolomic changes affecting the quality properties of fermented foods [100]. The two approaches were combined to study the structure and function of the core microbiota in Chinese soy sauce aroma type liquor production and facilitate understanding of the flavour development in the product as a two-stage process involving yeast initially for the production of ethanol, followed by a functional shift for the production of organic acids by the action of *Lactobacillus* [101]. De Filippis et al. [102] used a combinatorial approach involving meta-transcriptomic analysis to facilitate understanding of the ripening process of Italian cheese and possibilities for ripening acceleration. The obtained results indicated the roles of non-starter LAB in ripening-related activities and the temperature increase-related modulations on the microbiota structure and function during maturation for optimisation of production efficiency and product quality.

Although meta-transcriptomic analysis can provide valuable knowledge about the gene expression and potential functional activity, it might fail to establish direct associations between the microbiota and the environment, since the mRNA expression might not be directly associated with protein expression, and cell activity regulation occurs at the protein level. Therefore, the direct analysis of the proteins are essential as a complementary approach along with meta-transcriptomics to study the functional activity of the microbial community [103]. The meta-proteomic approach, which provides a large-scale study of the entire proteins expressed by a microbial community in an environmental sample, is useful for the identification and quantification of microbial activity at the post-translational level and could be the link between metagenomics studies and biological functions for understanding complex substrate-microbiome interactions [104,105]. Compared to other meta-omics approaches, meta-proteomic analysis is barely explored for studying food fermentation because of limitations, including the high cost, complexity of microbial samples, and the high similarity between many protein sequence reads [106,107]. Yang et al. provided a comprehensive review of the applications of proteomics in the study of fermented food and beverages [104].

## 5. Future Perspectives

The fermentation process has undergone significant improvement over the years. With technological advancement, it has become possible to manipulate the fermentation starter culture and the associated microbiome to standardise the product stability, improve sensory properties of the food, and ensure safety. Genome editing and microbiome engineering tools based on the CRISPR technology are evolving rapidly and are becoming highly efficient in improving microbial functionality. This modern technology is mostly applied in human cell research, and CRISPR/Cas9-based gene therapy has clinical potential. However, this technology still holds great promise for a wide range of applications, including in the food fermentation industry. The industry of fermented food and beverages may benefit significantly from the application of the revolutionary tools of CRISPR-based genome editing and microbiome manipulation, leading to improved control of the process and the properties of the end product by promoting the desired features linked to the sensory aspects, quality, shelf life, nutritional content, and safety of fermented food. This improvement would be coupled with increasing consumer acceptance and fewer governmental restrictions on the use of CRISPR/Cas9 gene editing in microbe manipulation, especially when no insertion of foreign DNA is involved. Scientific research is continuously proving the safety and precision of modern genomic manipulation tools, with no use of resistance markers and genetic stability of generated strains, thereby becoming accepted as a food-grade process.

However, it is important to highlight the worldwide popularity and acceptance of fermented food and its natural and artisanal nature. Therefore, spontaneous and traditional methods of preparation will continue to be a major part of the practice. Hence, the precise characterisation of the whole process and hygienic aspects are important to understand traditional fermentation and ensure the safety of consumption of fermented food. The available tools based on NGS technology and the rise of pioneering integrated multi-omics approaches have allowed deep understanding and high-resolution analysis of the fermentation process with many novel insight into the fermented-food microbiome and the role in the physicochemical and sensory properties of fermented food.

The advancement in tools available to study the fermentation process has revealed the inherited wisdom of ancient societies. The selection of suitable fermentation substrates to combine and set up the conditions of traditional fermentation led to the preparation of healthy products with improved sensory properties. The combined multi-omics approaches have provided cutting-edge discoveries in different microbiological research fields, while their application in studying fermentation is limited. Most studies use a single approach to study a particular aspect, especially because of the high cost of combining different omics approaches and the need for sophisticated bioinformatics and biostatistics skills for the analysis of such large datasets. A combined multi-omics approach would facilitate understanding the process, provide new systems-biology perspectives, and decipher the interaction among the fermentation microbiota, the substrate, and the environment. Overall, the near future will bring a greater range of applications of multi-omics in studying food fermentation, leading to detailed characterisation and efficient and sustainable production of fermented food and beverages.

## Figures and Tables

**Table 1:** Classification of the major types of fermentation related to food production.

Type	Biosynthetic Pathway	Responsible Microbes	Fermented Food
Lactic	Sugars are converted into lactic acid	Lactic acid bacteria	Yoghurt and kimchi
Acetic	Several substrates are converted into acetic acid	*Acetobacter*	Vinegar and water kefir
Alcoholic	Sugars are converted to alcohols and CO_2_	Yeast	Wine and beer
Alkali	Proteins are converted into amino acids, peptides, and ammonia	*Bacillus* and *Staphylococcus* spp.	Japanese nattu
